# Addressing Learner-Centred Barriers to Sexuality Education in Rural Areas of South Africa: Learners’ Perspectives on Promoting Sexual Health Outcomes

**DOI:** 10.1007/s13178-021-00651-1

**Published:** 2021-09-29

**Authors:** Ayobami Precious Adekola, Azwihangwisi Helen Mavhandu-Mudzusi

**Affiliations:** Department of Health Studies, College of Human Sciences, University of South Africa, Pretoria, South Africa

**Keywords:** Adolescents, Gender, Learner, Rural-based schools, Sexual and reproductive health, Sexuality education

## Abstract

**Introduction:**

The school-based sexuality education programmes in South Africa aim to improve the sexual and reproductive health of school-going adolescents. However, the high rate of unplanned pregnancy and sexually transmitted infections among learners in some schools in rural areas of King Cetshwayo district suggests that the programmes in these schools might not be effective due to certain learner-centred factors.

**Method:**

This qualitative study explored lived experiences of 84 learners from nine public schools in 2020 through focus group interviews. Data was analysed using Interpretative Phenomenological Analysis.

**Results:**

Learner-centred barriers to effective school-based sexuality education identified in this study were attitudes, age disparity, psychological status, peer pressure, socio-economic status, the exploratory attitude of learners, media, lack of role models, previous experiences, socio-economic status, and lack of parental love. These factors could reduce good sexual health. Learner-targeted interventions such as campaigns, using guest professionals, condom distribution, videos, on-site family planning, formal demonstrations, and on-site counselling could address these barriers.

**Conclusions:**

Addressing these barriers and implementing the proposed interventions will enhance school-based sexuality education and consequently improve adolescents’ sexual health.

**Policy Implications:**

The findings could guide programming, implementation, and delivery of school-based sexuality education leading to improved adolescents’ sexual and reproductive health.

## Introduction

An effective school-based sexuality education programme promotes a safe learning environment that accommodates gender equity and equips adolescents with knowledge and skills that empower them to make informed decisions about their sexuality (Ogolla & Ondia, 2019). However, risky sexual practices and high numbers of reported teenage pregnancy among learners in the rural areas indicate that there are factors that reduce the effectiveness of sexuality education in these communities. In South Africa, Du Preez et al. (2019) note that the prevalence of unplanned pregnancy among learners remains alarmingly high despite the implementation of school-based sexuality education. Besides a high rate of teenage pregnancy, the South African National AIDS Council (SANAC) (2017) reports that 37% of new HIV infections occur among school-going adolescent girls. This suggests that learners might be engaging in risky sexual practices that make them vulnerable to STIs, including HIV (Department of Basic Education (DBE), 2016). Another report indicates that most pregnant learners were from schools in rural areas (Statistics South Africa, 2016). While there is a large body of evidence that well-implemented school-based sexuality education programmes can promote positive sexual and reproductive health (SRH) outcomes among young people (Le Mat et al., 2020; Panchaud et al., 2019; Ponzetti, 2016; Tanton et al., 2015), the rising number of pregnant learners in rural areas of King Cetshwayo district (Office of the Premier, KwaZulu-Natal, 2020) suggests sexuality education programmes might not be effective due to certain factors in these communities. This prompted the researchers to conduct a study that explored learners’ lived experiences of sexuality education offered in these schools. This study identified, described, and interpreted learner-centred determinants that influence school-based sexuality education.

Sommer and Mmari (2015) define determinants as the conditions in which individuals are born, grow up, and work. These could be circumstances shaped by family, community, power, and access to resources at various levels that influence the effectiveness of sexuality education programmes. Contextual and interrelated factors such as family background, culture, religion, community, poverty, peers and societal influence affect adolescents’ SRH, and wellbeing are targeted by sexuality education. Viner et al. (2012) concur that these factors shape young people’s choices and chances to live healthy lives. This line of thought is supported by Khuzwayo and Taylor (2018), who assert that the microsystem within which an individual functions exerts significant influence on their efficacy and how they react to SRH education. In the same vein, Haberland and Rogow (2015) posit that an individual’s social environment has a significant influence on their sexual health and rights. A review of the literature revealed that these determinants may hinder the effectiveness of school-based sexuality education programmes.

## Social Determinants Hindering the Effectiveness of School-Based Sexuality Education Programmes

It emerged from the literature review that culture, parental opinions, religious background, poverty, lack of access to SRH services, school-related factors, and substance abuse can limit the effectiveness of school-based sexuality education and its influence on adolescents’ SRH outcomes.

### Poverty

While one of the main purposes of school-based sexuality education is to empower adolescents to access SRH services, a study by Sommer and Mmari (2015) indicates that poverty hinders young people from doing this. Handa et al. (2017) assert that poverty fosters early sexual debuts and transactional sex among young people. A study conducted in a rural district of South Africa by Lambani (2015) revealed that 80% of pregnant teenage participants reported they engaged in unprotected sex because they were poor. Likewise, Ward et al. (2015) state that poverty imposes severe limitations on parents’ ability to influence their children positively, because poor parents cannot provide food and necessities for their children. These findings are corroborated by Khuzwayo and Taylor (2018), who report that young people choose their sex partners strategically for material and monetary gain. Therefore, poverty may increase risky sexual practices among school-going adolescents, thus undermining the key objectives of sexuality education programmes.

### Parental Opinions

Wekesah et al. (2019) argue that parental opposition to key messages of sexuality education constitutes a significant challenge to its effectiveness. This is consistent with the findings of Van Wesenbeeck et al. (2016), who caution that efforts by teachers to empower young people with information and skills to help them achieve sexual health and wellbeing may fail if they receive conflicting messages at home. While parents are likely to support sexuality education that aligns with their cultural values and beliefs, it could undermine teachers’ fidelity to the sexuality education curriculum because they may skip some key aspects to avoid parental criticism (Wekesah et al., 2019). The same authors maintain that parents’ educational status may influence whether they accept the messages of school-based sexuality education programmes or not, because rural residents who are less educated are more inclined to reject sexuality education messages that contradict their cultural values and practices.

### Cultural Norms

Cultural norms are prevalent customs, practices, and characteristics in a community (Khuzwayo & Taylor, 2018). These norms and practices can influence the effectiveness of school-based sexuality education programmes and adolescents’ SRH outcomes (Beyers, 2011). A South African study revealed that cultural norms could influence adolescents’ sexual behaviour negatively (Khuzwayo & Taylor, 2018). In addition, cultural norms are known triggers of moral panic, which may affect every aspect of implementing sexuality education in a community (UNESCO, 2019a). The key concepts of sexuality education are gender equality, human rights, violence, and access to SRH services by the community actors. A study by Ndinda et al. (2017) elucidates how cultural norms act as a barrier to accessing and using SRH services such as contraceptives in the rural areas of South Africa. Furthermore, Francis (2013) indicates that sexuality education teachers feel pressure to promote certain values and moral positions acceptable to the community. A study conducted in Uganda revealed that the cultural perception that sexuality education encourages sexual activity and immorality among young people triggered strong resistance to its implementation by the government and educators (Ninsiima et al., 2019). In a study conducted in Kenya, Wanje et al. (2017) reveal that cultural norms can impede sexuality-related discussions between school-going adolescents and adults. Besides hindering the effectiveness of sexuality education for learners, Glover and Macleod (2016) concur that sexuality education concepts that contradict educators’ socio-cultural values make them uncomfortable, thereby affecting their ability to teach sexuality education lessons effectively.

Beyers (2011) argues that learners place higher value on knowledge obtained from cultural practices than on sexuality education information provided by teachers. Furthermore, certain acceptable cultural norms like having multiple sexual partners and testing female fertility through unprotected sex inadvertently promote risky sexual behaviour in young people, thus undermining the key messages of sexuality education (Khuzwayo & Taylor, 2018). This is supported by a recent study which indicated that cultural practices in some communities perpetuate gender inequality, which limits school-going adolescent girls’ ability to negotiate and make decisions; they may therefore have unhealthy sexual relationships (Ninsiima et al., 2019). Such gender power imbalance fosters gender-based violence, unprotected sex, and multiple sexual partners among young people (Khuzwayo & Taylor, 2018).

### Religion

According to Olowu (2015), the religious beliefs of a particular community are likely to shape socio-cultural norms and attitudes towards school-based sexuality education programmes. Similarly, the teaching of some religious organisations triggers distrust of key messages of the school-based sexuality education curriculum like contraception, gender diversity, and gender equality, which are offensive to their beliefs and doctrine. Such teachings may promote stigmatisation of and discrimination against people living with HIV (PLHIV) and lesbian, gay, bisexual, transgender, and queer (LGBTQ) people (Armstrong-Mensah et al., 2019; Olowu, 2015).

A study by Olowu (2015) shows that some religious groups dissuade PLHIV people from adhering to prescribed anti-retroviral therapy and promote prayer as a cure for HIV. In a study conducted in the USA, Ponzetti (2016) reports that certain religious leaders propagate rigid gender concepts in addition to portraying any non-heterosexual and non-marital sexual activities as sins against God. Influential religious groups can also mobilise societal resistance to the implementation of school-based sexuality education, as was the case in Uganda where policymakers were pressured to remove curriculum contents that were deemed offensive to their religious beliefs (Ninsiima et al., 2019). Such religious stances and practices hinder young people from accessing SRH services and undermine the effectiveness of school-based sexuality education. Wekesah et al. (2019) argue that contradictory messages from religious groups undermine key sexuality information received by learners and put pressure on teachers to promote sexual abstinence over other contraception methods and avoid sensitive topics like condoms, abortion, gender equality, gender diversity, and sexual identity in lessons.

### Substance Abuse

The tendency of young people to experiment and engage in risky behaviours usually increases at adolescence (Francis et al., 2019). This is corroborated by Albert-Lorincz et al. (2019), who indicate that such risky behaviours include experimentation with substance use. The Center for Disease Control and Prevention (CDC) (2018) warns that regular use of drugs such as opiates, ecstasy, alcohol, and other illegal substances negatively affects the health and wellbeing of the users. A study by Ritchwood et al. (2015) revealed a higher prevalence of risky sexual practices and early sexual debut among adolescents who regularly abuse substances compared to those who do not. This is supported by other studies which affirm that adolescents who use mind-altering substances are likely to engage in risky sexual practices leading to adverse SRH outcomes (Khadr et al., 2016; Muche et al., 2017). A similar conclusion, that drug use promotes risky sexual behaviours among adolescents, was reached by several other studies conducted in Ghana, Iran and South Africa (Doku, 2012; Francis et al., 2019; Yazdi-Feyzabadi et al., 2019). These studies highlight the need for a sustainable plan to address adolescents’ substance abuse to enhance the effectiveness of sexuality education.

### Lack of Access to Sexual and Reproductive Health Facilities and Services

A report by the DBE (2017) revealed that lacking access to friendly, non-judgmental SRH services is a barrier to effective sexuality education for young people. According to Yakubu and Salisu (2018), sex-related stigma in community health facilities further contributes to low adolescent uptake of contraceptives and other sexual health services. This agrees with an earlier report by Haberland and Rogow (2015), which emphasises the need to link friendly SRH services with school-based sexuality education programmes to strengthen their effectiveness.

### School-Related Factors

The availability of appropriate teaching and learning resources at school level is a critical determinant of school-based sexuality education programmes (Iyer et al., 2014). A lack of relevant resources coupled with educator-related factors such as lack of competence and cultural and religious views constitute a barrier to effective implementation of sexuality education programmes (Bonjour & Van der Vlugt, 2018). Glover and Macleod (2016) reflect that sexuality education teachers struggle with certain topics like sexual diversity, mainly due to lack of proper training or because it conflicts with their socio-cultural values. In addition, Smith and Harrison (2013) say that sexuality education teachers would prefer using invited speakers in schools for some topics because of their own perceived lack of expertise and learners’ fatigue with their handling of these topics. This is in line with the findings of Bonjour and Van der Vlugt (2018), who report that young people rate their sexuality educators’ handling of topics poorly. The reviewed literature indicated that continuous in-service training of teachers is crucial to the success of any school-based sexuality education programme (Haberland & Rogow, 2015; Ogolla & Ondia, 2019). Such training should enhance teachers’ pedagogical competency and fidelity to curricula implementation strategies.

While the reviewed literature addresses social determinants that are community- and educator-centred, this study aimed to use learners’ experiences and perspectives to describe and address learner-centred factors that could limit the positive impact of school-based sexuality education programmes on adolescents’ SRH.

## Methods

### Design

This phenomenological study used the experiences and perspectives of adolescents to describe the determinants of school-based sexuality education in the rural areas of King Cetshwayo district. Polit and Beck (2017) argue that this design empowers researchers to gain deep insight into the shared experiences of the participants and to have a better understanding of the contexts in which these experiences occurred. The researcher asked participants critical and clarifying questions about sexuality education programmes in their schools. Thereafter, the researcher listened to and recorded participants’ self-described perspectives and experiences.

### Setting

The study was conducted in rural areas of King Cetshwayo district in KwaZulu-Natal province. Nine public secondary schools that offer sexuality education as part of the mandatory subject Life Orientation were selected.

### Sampling

The researcher used purposive sampling to recruit Grade 10 and 11 secondary school learners to participate in the study. The research was conducted in 2020. The inclusion criteria were as follows: be a registered Grade 10 or Grade 11 learner in a school in the rural areas of King Cetshwayo district, be between 14 and 19 years old, and reside in the study area. Other inclusion criteria were fluency in either or both English and IsiZulu, willingness to be audio recorded, readiness to sign informed consent, and signed parental consent to participate in the study.

A total of 84 participants (49 girls and 35 boys) took part in this study. While the initial 84 participants were sufficient and there was no need for further recruitment of participants, the researcher was also guided by data saturation, which occurs when new information obtained from participants is a repetition of data obtained previously (Saunders et al., 2018). This implies that the researcher cannot develop new ideas and themes by collecting additional data. Mavhandu-Mudzusi (2016) explains that data saturation occurs when data collected from participants does not yield new insights which are relevant to the objectives of the study.

About 58% (*n* = 49) of the participants reported that they were sexually active; about 84% (*n* = 41) reported regular condom use, and about 59% (*n* = 29) of the sexually active participants claimed to have been involved in multiple sexual relationships in the past three years. Four female participants reported that they had been pregnant before and 25% (*n* = 21) reported they had no previous exposure to alcohol at the time of data collection.

### Data Collection

The researcher conducted nine focus group interviews from March to July 2020. A focus group interview guide which was developed by the researcher and refined in a pilot study was used to mediate the interview process. The focus group interviews took place at the premises of the selected schools. Guided by Kvale’s (1996) interview guidelines, cited in Qu and Dumay (2011), the researcher asked the participants central questions such as: “Describe your experience and perceptions as a learner of the sexuality education offered at this school.” As the mediator of the interview process, the researcher probed and prompted participants where necessary to obtain rich and comprehensive data.

Each interview session was audio recorded and lasted for about 120 min. While some data was collected after the COVID-19 lockdown was relaxed, the researcher ensured that DBE COVID-19 safety protocols such as social distancing, hand sanitising, wearing facemasks, and using well-ventilated venues were strictly adhered to during each focus group interview. In addition, to enhance the audibility of the participants who were wearing masks, the researcher used a high-quality audio recorder. The researcher observed and captured participants’ non-verbal cues and recorded his own reflections on the data collection process in field notes. The collection of data and its analysis were iteratively done until data saturation was reached.

### Data Analysis

The researcher transcribed the audio-recorded data from each focus group interview verbatim within 2 days into Microsoft Word. Guided by Noon (2018), the researcher independently analysed each of nine transcripts of the interviews using Interpretative Phenomenological Analysis (IPA) data analysis framework. The researcher read and re-read transcribed data and field notes while listening to the recorded audio of the interview. The researcher made notes regarding his reflections, observations, and thoughts relating to the participants’ narratives. Connections between emerged themes were identified, and related themes were categorised into superordinate themes. In addition, an expert independent IPA coder was engaged to analyse the transcribed data independently. The themes that emerged were compared, resulting in the final table of themes with two superordinate themes, several sub-themes, and relevant quotes from the participants.

### Trustworthiness

The researcher followed Lincoln and Guba’s (1985) criteria to ensure the trustworthiness of the study. These criteria are transferability, dependability, confirmability, and credibility. The researcher carried out ongoing member checking to ensure credibility; to verify that participants’ perspectives and experiences were captured accurately, they were allowed to listen to the recordings and given the transcripts to confirm that their views were reflected accurately.

Additionally, the researcher enhanced the confirmability and dependability of the research by using field notes to document relevant information about settings such as times, dates and locations. The researcher’s colleagues and the expert coder independently transcribed the recorded data verbatim. In the same vein, the independent coder analysed the transcribed data and deduced independent themes. While the researcher kept an audit trail of all research activities, emerging themes found by both the researchers and the independent coder were compared to enhance the confirmability of the study. The researcher provided thick descriptions and rich details of the research context and participants’ demographic data to ensure transferability, which was strengthened by detailed descriptions of the study setting, sample, and research process. Likewise, the researcher provided robust descriptions of his assumptions and experiences during data collection to further enhance the transferability of the study.

### Ethical Considerations

The University of South Africa and the KwaZulu-Natal provincial DBE granted ethical approval to conduct the research. Besides emphasising the participants’ rights of refusal and assuring them that their participation was voluntary, the researcher explained the nature, purpose, and potential benefits of the study to them. The participants were told they could withdraw from the study at any time without incurring any negative consequences. The researcher collected all completed and signed informed consent letters from the parents/guardians and consent forms from the participants before data collection commenced.

To protect the identities of the participants, pseudonyms were used in the transcripts and in reporting the data. All the focus group interviews took place in suitable, convenient, and comfortable venues to ensure confidentiality. To maintain anonymity during focus group interviews, the researcher adopted Sim and Waterfield’s (2019) approach, notifying the participants of the public nature of group interviews and the attendant anonymity issues. He also explained the importance of anonymity and how participants’ full cooperation was needed to meet this ethical requirement. Thereafter, the researcher asked participants who were unsure about the issue of anonymity to withdraw before the focus group interviews started. He also ascertained that all participants understood their responsibilities regarding anonymity.

Since some of the focus group interviews took place during the COVID-19 pandemic, the researcher ensured strict compliance with COVID-19 safety protocols: using well-ventilated venues, maintaining a two-metre distance between participants, providing 70% alcohol-based hand sanitisers, and wearing facemasks. The researcher kept all data safely in a secure electronic folder to prevent access by unauthorised persons.

## Results

The analysis of the data revealed prevailing learner-centred factors that are likely to hinder the impact of school-based sexuality education programmes on adolescents’ SRH. In addition, there were learner-targeted interventions that could mitigate these barriers. The themes that emerged were categorised as learner-centred barriers to effective school-based sexuality education and learner-targeted interventions to enhance school-based sexuality education programmes. Each theme has several sub-themes.

### Learner-Centred Barriers to Effective School-Based Sexuality Education Programmes

This theme discusses learner-related factors that inhibit the effectiveness of sexuality education, based on the experiences and perspectives of the study participants.

### Age

The age of young people could hinder effective communication about sexuality between them and their educators or parents. Some participants felt the contents of the sexuality education curriculum in their schools were not appropriate for their ages, while others felt uncomfortable discussing sex-related topics with adults because they accepted their parents’ view that they were too young for such discussions.

I’m too young not in that way, my parents even say to me ‘you are too young for those things you are talking about.’ We are regarded as still too young to learn about sex. (Wandi, female, 17 years old)

It is not comfortable because we are young and, in most cases, we are not that mature to learn those things at school because we mostly think that we are jumping, like we are, I don’t know how to put it, but it feels like we are not brought up to discuss sex with our elders. (Sisanda, female, 17 years old)

Rejecting sexuality education messages due to age constitutes a barrier to school-based sexuality education’s effectiveness. Besides their age, the results showed that the psychological status of school-going adolescents could limit the impact of sexuality education on their SRH.

### Psychological Status

Some participants mentioned that their psychological state influenced their acceptance or rejection of knowledge acquired during sexuality education lessons:

Sometimes it depends; life is tough in this community, so we do things when we are under pressure or stress. To keep the stress out of ourselves, we do things we should not normally do to forget about our stress. When you are stressed, you may not even remember what you learnt in the class. You just want to feel good, and that why we do such things like smoking, drugs, and sex to forget. (Mthokosizi, male, 17 years old)

### Attitudes

The findings revealed that negative attitudes in learners could upset the learning environment, thus making sexuality education lessons uncomfortable for other learners and educators. Some participants indicated that lack of respect, teasing, bullying, and mocking exhibited by some learners could affect the effectiveness of sexuality education.

When it comes to sexual intercourse and sexuality education, we are scared to express our views out, because others may perceive that we are not well behaved. Some people tease you if you are honest during class discussion. So, I am not comfortable discussing sexuality education in the class because I don’t want to deal with such issues and gossip after the lesson. Other learners at school know that I was once pregnant, and so whenever we are talking about learners’ pregnancy or sexual intercourse, they would pick on me, make jokes to each other, and laugh. (Zamile, female, 17 years old)

There is a lack of respect for the teachers by some learners, and that is why they do not do what they learnt in sexuality education. (Fezeka, female, 16 years old)

### Peer Pressure

It emerged that young people’s urge to measure up to their peers’ expectations could inhibit the effectiveness of sexuality education in their schools.

If everyone speaks about it that they have done it, so you might feel you have to do it too. Peer pressure makes you believe that you should do what everyone else has to do if you want to fit in. If you’re the only one in your group of friends who is not sexually active, you will be pressurised into sexual intercourse. (Nomfundo, female, 17 years old)

### Socio-economic Status

Apart from peer pressure, the socio-economic status of learners could be a barrier to sexuality education effectiveness. One participant had this to say:

Poverty makes boys and girls end up selling their bodies in exchange for money to provide for their basic needs. Blessers seduce girls with money, saying if they have sexual intercourse with them, they will give them money, and a girl from a poor background would accept this. Similar things happen to the boys, too, in the community. (Mzomuhle, Male, 17 years old)

### Exploratory Attitude

In addition to the socio-economic status of learners, the study indicated that adolescents’ curiosity pushes them to experiment sexually despite what they learned in sexuality education lessons.

We are teenagers who like to have experience with these things. Our teachers and our parents probably did it when they were teenagers too. Sometimes, we do not see ‘experimenting’ with friends as a wrong thing because many young people do that in the community. (Andile, female, 17 years old)

### Lack of Positive Role Models

Besides an exploratory attitude, participants mentioned that lacking positive role models could be a barrier to accepting sexuality education messages.

Learners engage in sexual intercourse even when they are underage because they see their parents and people in the community doing these stuff. (Osiphayo, female, 17 years old)

Some learners just want to be like their parents who sleep around with men or women, and this has nothing to do with what they were taught in LO. Even in school, some teachers do that, and some learners may want to be like them and sleep around. (Qiniso, male, 17 years old)

### House Parties

Another learner-centred barrier to effective sexuality education was widespread adult-hosted house parties in the community. The findings revealed that older men frequently organised house parties to entice girls and boys into sexual activities.

We also have a house party organised by the adults for youngsters to get intoxicated with alcohol, which results in sexual intercourses that often lead to teenage pregnancy. House parties are the main reason teenagers do illegal stuff like drinking, smoking, and having sex. (Siphesihle, female, 16 years old)

### Media

It emerged that exposure to certain media could negatively influence the outcomes of school-based sexuality education.

We want to experience what we see on media rather than what we learn about sexuality. Many learners want to do the things they see on TV. They want to consume alcohol and drugs and have sex. (Zama, female, 16 years old)

### Personality

The findings suggested that the personality traits of learners could act as a barrier to the effectiveness of sexuality education.

I am not the type of person that is open and comfortable during Life Orientation lessons. I’m shy, and I am introvert. In the class, I am not comfortable hearing about sexuality education in front of males, that’s why I do not like to discuss sex. (Zonke, female, 17 years old)

### Lack of Parental Love

Participants reasoned that adolescent girls are likely to engage in risky sexual practices if they lack parental love at home.

Some girls may be lacking love from family members. Parents may not be giving them attention and love they deserve, and so they look for love from boys that could lead to risky sexual activities even though they have been taught about it. (Sibonile, female, 17 years old)

### Lack of Recreational Facilities

Besides lack of parental love, a lack of after-school recreational facilities in the community increases the risk of negative SRH outcomes.

No recreation after school and learners hope they get invited to house party somewhere that may lead to risky sexual practices. Learners use sex to keep themselves entertained because there are no other forms of entertainments for them to deal with boredom. (Seneme, female, 17 years old)

### Experience

Some participants opined that the previous experiences of learners could shape their attitude to sexuality education programmes in their schools.

Some learners who were rape victims can be uncomfortable during lessons on sexual violence without teachers knowing about it, only people close to them know this because they stay quiet. Others may have their reasons for not being comfortable. (Sthembiso, male, 18 years old)

### Child-Support Grants

Some participants felt that the monthly child support grant is a motivation for girls from poor backgrounds to become pregnant. This implies that girls who want to benefit from a child-support grant could disregard messages on safe sexual practices.

Some girls tend to have sex to become pregnant so that they will get a grant from the government. They do this because they are poor. (Thabani, male, 17 years old)

### Substance Abuse

Learners’ use of mind-altering substances could negatively impact the effectiveness of sexuality education. One participant said:

I think that substance abuse is one of the issues that leads to teenage pregnancy. When you use drugs, alcohol or something, you cannot control yourself; you can do just anything because of the substance abuse. (Samkelo, female, 17 years old)

Besides the learner-centred barriers that limit the impact of sexuality education on adolescents’ SRH, participants proposed interventions that could enhance the effectiveness of school-based sexuality education programmes.

### Learner-Targeted Interventions to Enhance School-Based Sexuality Education Programmes

This theme focuses on proposed learner-targeted interventions aimed to enhance the effectiveness of sexuality education in schools. All the proposed interventions emerged from the data collected from the participants. UNESCO (2019b) recommends that young people should be involved at every level of school-based sexuality education programming. The proposed learner-targeted interventions include awareness campaigns, guest health care professionals, on-site condom distribution, video shows, family planning at school, formal demonstrations, and on-site counselling services, in addition, sport activities, social media use, multi-stakeholder collaboration, help clubs, guidance by community leaders, and advocacy.

### Campaigns

To mobilise stakeholders on SRH issues faced by adolescents, participants suggested an SRH awareness campaign that addresses misconceptions about school-based sexuality education and secures their support in addressing known adolescents’ SRH issues.

We need campaigns from different people. They can come together, work together in creating a campaign that could teach young people as to how should protect themselves in order not to fall pregnant at a young age. (Luyanda, male, 17 years old)

### Guest Health Care Professionals

Besides SRH awareness campaigns, using skilled health care professionals as guest teachers is a suggestion by participants to enhance sexuality education effectiveness.

We can welcome people who are more educated in sexual education at least twice a year to give speeches or talk about it. Organise visits by healthcare workers to talk to learners during LO lessons and address sexuality issues. (Nomfundo, female, 17 years old)

### Condom Distribution at School

There is a lack of friendly access to condoms. Participants believed that distributing condoms to learners would strengthen sexuality education and promote their SRH.

School should offer condoms in case learners do become sexually active, and they need to be protected. Condoms should be given out to everyone just to be safe. It is not easy to get condoms around. Also, teach more on how to use condoms properly. (Luvuyo, male, 16 years old)

### Video Show

The use of videos to deliver sexuality education is another intervention proposed by participants. They felt that using videos in lessons would help them to understand the concepts being discussed better.

Showing us videos will help us to have a better understanding of sexuality education. (Ntenga, male, 17 years old)

### Family Planning Services at Schools

In addition to using videos as teaching aids in the classroom, participants suggested introducing on-site family planning services in schools to address the lack of accessible, convenient, non-judgmental and youth-friendly SRH services.

Distribute morning-after pills to girls and other things like implants, injections name them. We can’t stop learners from having sex, but we can help them to protect themselves. Distribute them in school so learners can have protected sex or mobile clinics should come to schools each month. That’s likely going to reduce teenage pregnancy and make it effective. (Qiniso, male, 17 years old)

### Formal Demonstration

Talking to young people without demonstrating the message may not provide them with the skills they need to make the desired behavioural changes. Participants wanted sexuality education topics to be communicated in practical ways that would improve their comprehension and equip them with necessary skills.

No experimentation is being done by teachers, they need to show us. Things that we learnt we don’t know how to do them. Teachers must show us how it is done to make it more effective. Instead of just telling us, they should show us. (Ayanda, female, 17 years old)

### On-Site Counselling Services

Some participants proposed that their schools should have an on-site counselling service manned by qualified counsellors rather than their Life Orientation educators.

The school needs supportive and relatable guidance and counsellor that is appealing to learners and know how to give good advice to us when we need it. Advise us about the actual truth about teenage pregnancies and the difficult life ahead not false stories. (Nosipho, female, 17 years old)

### Sport and Entertainment

Another learner-targeted intervention that emerged is providing recreational facilities for after-school sport and entertainment. Participants felt that access to these facilities would engage them productively and promote positive SRH outcomes.

Our communities must keep us as teenagers busy, like having sports or participating more in sports and having some small events teaching us about the important life skills and rewards. We need recreational facilities in our community that will keep us busy after school. (Nompilo, female, 18 years old)

### Use of Social Media

The study findings revealed that using digital platforms such as social media could strengthen school-based sexuality education due to the proliferation of mobile digital devices and the popularity of social media platforms among young people.

Using WhatsApp group to connect LO teachers and parents to share information could help. We are always on social media and all that, so we could use social media to let people, especially young people, know about sexuality. (Thando, male, 18 years old)

### Forming Help Clubs

The formation of help clubs to generate social capital in the community is another proposed intervention that emerged from the study. Participants argued that help clubs would strengthen the commitment of young people to practise what they learn in sexuality education lessons.

I think in our community we need to form “Help Club” to help one another. In our community, we need to be our brothers and sisters’ keepers. We need to influence one another positively to make the right decisions. Also, outside the school, we need to remind ourselves of what we learnt about sexuality education at school. (Wandi, female, 17 years old)

### Multi-stakeholder Collaboration

Participants felt that educators, parents, community actors, and religious groups should work together to improve the impact of sexuality education in the community.

If parents and teachers work together, we can improve the effectiveness of sexuality education in our community. Maybe we can reduce teenage pregnancy, STI cases and HIV among learners in the community if our parents, churches and community leaders get involved. School can also talk to our parents at least once a year to make it comfortable at home to speak to our parents about it. (Asande, female, 17 years old)

### Guidance by Community Leaders

Using trained community leaders to counsel young people about sex may address misconceptions about sexuality education and promote positive SRH outcomes among adolescents in the communities.

In order to make the sexuality education work, I think the community leaders should be our other counsellors. (Luyanda, male, 17 years old)

### Advocacy

Participants recommend using influential people and opinion leaders to promote the messages of the sexuality education curriculum among young people.

I think there should be event days that talk to youths about sexual life and in those events, they should invite famous people because as we are teenagers, we like famous people. We look up to them as our role models, and we do everything that they do. If we see the famous person telling us about the impact, it has in our lives maybe we can just listen to them because we look up to them as our role model. (Nompilo, female, 18 years old)

## Discussion

### Learner-Centred Barriers

The impact of sexuality education on adolescents’ SRH is restricted by learner-related factors such as age, psychological status, an exploratory attitude, peer pressure, socio-economic status, lack of role models, house parties, media, personalities, lack of parental love, lack of recreational facilities, past experiences, child support grants, and substance abuse. These findings agree with several studies which indicate that the age difference between young people and their teachers or parents could hinder effective communication on sexuality-related topics both in schools and at home (Anyanwu et al., 2020; Motsomi et al., 2016; Pariera, 2016).

The psychological state of young people is another determinant of SRH outcomes. The study findings concur with the results of Anatale & Kelly’s, 2015 study, which revealed that young people with symptoms of depression are more likely to engage in harmful sexual activities. The attitudes of learners to sexuality education, coupled with their need to explore, could negatively impact sexuality education outcomes. This is supported by earlier studies which report that some learners did not take sexuality education lessons seriously and others were uncomfortable during lessons because of teasing from other learners regarding their sexual orientation and virginity (Bonjour & Van der Vlugt, 2018; Glover & Macleod, 2016). These studies maintain that learners’ negative attitudes could make others uncomfortable and upset the teaching and learning environment during sexuality education lessons.

In the same vein, the study showed young people’s curiosity and need to experiment could influence them to ignore SRH messages. This is line with the 2019 study by Francis et al., who note that adolescents’ tendency to explore their sexuality usually leads to increased risky behaviour. Similarly, a large body of evidence suggests that adolescents’ exploratory attitude to sex could affect SRH outcomes negatively (Albert-Lorincz et al., 2019; Cooper et al., 2015; Bonjour & Van der Vlugt, 2018). However, learners’ attitudes toward school-based sexuality education could also have a positive influence on SRH outcomes (Ozuri & Akarah, 2016).

While sexuality education programmes aim to equip adolescents to challenge harmful peer pressure (UNESCO, 2018), it emerged that young people might not apply acquired sexuality knowledge due to peer pressure. This is consistent with the CDC’s 2018 report, which indicates that peer pressure could be a threat to adolescent decision-making. Cooper et al. (2015) say that peer influence is a significant factor that promotes adolescents’ sexual risk-taking. This aligns with the findings of Yakubu and Salisu (2018), who identify peer pressure as a barrier to effective school-based sexuality education in Sub-Saharan Africa. This suggests that any intervention to improve school-based sexuality education must address negative peer influence and seek to enhance young people’s decision-making skills.

Learners with a poor socio-economic background are likely to engage in risky sexual activities such as transactional and intergenerational sex to negotiate themselves out of difficult financial situations, regardless of knowledge acquired in school. This finding agrees with other studies that highlight poverty as a key contributor to negative SRH outcomes (Pop & Rusu, 2015; Willan, 2013). In the same vein, Ward et al., 2015 study conducted in South Africa affirms that the economic background of young people could influence their sexual behaviour. This conclusion is supported by Handa et al., 2017 finding that poverty is a driver of adolescents’ early sexual debut and transactional sex in Kenya. In addition, the sexual exposure that learners from poor homes experience daily could undermine the key SRH messages of school-based sexuality education programmes (Wood, 2013).

Learners unwittingly or intentionally learn certain habits from their parents, teachers, and community leaders, who may be their role models. The study found that the disposition of these role models influences the choices of young people in the community. The lack of positive role models could also undermine the effectiveness of school-based sexuality education. This is in line with the findings of Johnson et al. (2016), who report that young people develop their character and make their choices by looking up to their role models. Khuzwayo and Taylor (2018) also argue that when their role models and parents have many sexual partners, many adolescents will embrace and engage in risky sexual practices. A study by Hurd et al. (2009) concurs that lack of positive role models for adolescents is associated with negative SRH outcomes.

Some adults in the community regularly organise house parties to lure learners into risky sexual activities. This could weaken the impact of sexuality education on adolescents’ SRH, as young people could disregard knowledge acquired in lessons due to their vulnerability. Learners who attend these parties are exposed to mind-altering substances, making them vulnerable to risky sexual activities. This finding aligns with the report of Hittner et al. (2016), who assert that frequent unprotected sex and alcohol abuse among adolescents is associated with party attendance. Similarly, several studies agree that party attendance among adolescents is correlated with high-risk sexual behaviour (Bersamin et al., 2012; Okafor et al., 2018).

The exposure of young people to sexualised media that is inappropriate for their age is another barrier to effective sexuality education. This is supported by Lin et al. (2020), who posit that exposing adolescents to sexually explicit media is strongly related to risky and harmful sexual behaviour like unprotected sex and early sexual debut. Lanre-Babalola (2018) notes that media could be a tool to disseminate positive SRH messages among young people, but cautions that adolescents’ exposure to sexual content on media promotes risky sexual practices. Likewise, other studies highlighted the inherent dangers of adolescent exposure and access to sexualised media contents to their sexual health (Johnson & Bridges, 2018; Oladeji & Ayangunna, 2017).

The results indicate that the personalities of school-going adolescents could be a barrier to effective sexuality education. Young people’s personalities play a significant role in their learning (Luqman & Khalid, 2018). Furthermore, in a study conducted in Poland, Kurpisz et al. (2016) report that adolescents’ personality traits moderately influence their sexual behaviour and outcomes. Van Leeuwen and Mace (2016) clarify that different personality traits among young people are correlated with varying SRH outcomes. In line with the literature review, which showed that parental love is a determinant of adolescent SRH (Mmari et al., 2016; Sentino et al., 2018), the findings of this study reveal that lack of parental love pushed some young people to engage in risky sexual practices despite the information provided in class during sexuality education.

Recreational and sporting facilities could foster an environment that protects young people from risky sexual practices (De Wet et al., 2018). The lack of recreational facilities encourages risky sexual behaviour in adolescents who seek to relieve boredom after school hours. This finding is consistent with Akers et al. (2011) study, which shows that lack of access to neighbourhood recreational and sporting facilities by young people promotes risky sexual engagement. These authors maintain that adolescents who have access to sporting and recreational facilities or who are involved in community-based recreational activities are less likely to engage in risky sexual behaviour leading to adverse SRH outcomes.

The findings indicate that past negative sexual experiences could also inhibit the effectiveness of sexuality education if left unaddressed. An earlier study by Pringle et al. (2017) agrees that adolescents’ past sexual experiences could affect their personal attitude to sexuality and their sexual behaviour. This is reinforced by Castro et al. (2019), who indicate that young people who were sexually abused as children are likely to have an earlier sexual debut, multiple sexual partners, and engage in other harmful sexual practices.

It also emerged that monthly child-support grants could be a motivation to disregard SRH messages because adolescent girls want to receive this money by having children. While an earlier study by Mbulaheni et al. (2014) reports that the child support grant is a driver of teenage pregnancy in rural areas of South Africa, a more recent one by Ngubane and Maharaj (2018) disagrees that a child support grant encourages teenage pregnancy in rural areas of South Africa.

Our results showed that substance abuse among adolescents could increase earlier sexual debut and harmful sexual engagement. There is a large body of evidence that support this conclusion (Ritchwood et al., 2015; Muche et al., 2017; Guzmán & Stritto, 2012). Furthermore, the study by Du Preez et al. (2019) agrees that substance abuse among adolescents is a driver of teenage pregnancy. Therefore, access to mind-altering substances and their subsequent use by young people could lead to negative SRH outcomes.

### Learner-Targeted Interventions

Campaigns to create awareness of how school-based sexuality education enhances adolescents’ SRH could shape the perceptions and opinions of key stakeholders. These campaigns could generate support for sexuality education and promote its acceptance in the community, creating a favourable and supportive environment for young people to practise what they learn in sexuality education lessons. Brug et al. (2011) note that awareness campaigns are essential for health promotion interventions targeting a particular population. Various health promotion campaigns such as immunisation, promotion of safe sexual practices, and prescribed medication adherence have been successfully used to enhance health outcomes in targeted populations (Kaur et al., 2015). This is supported by Cruz et al. (2013), who recommend that innovative materials should be used in such campaigns to enhance effective communication about the subject of interest. Periodic school visits by health care professionals is another proposed intervention that could enhance the impact of sexuality education on learners’ SRH. While the visits by these professionals may be insufficient in themselves, they could provide support for learners and teachers alike on certain topics they might not be comfortable with due to their culture, community norms, lack of skills and self-censorship (Goldman, 2011). This is in line with the recommendation of Pound et al. (2017), who report that many young people welcome and support visits by sexual health experts to address them on sexuality education in their schools because these people were perceived as less judgemental, more competent, and able to provide more confidentiality than their teachers.

While the DBE’s policy promotes discreet access to condoms for learners who meet the age requirement (DBE, 2017), the participants reported lack of access to condoms and proposed they should be distributed at school to enhance the effectiveness of sexuality education. This recommendation agrees with the findings of Wang et al. (2018), who argue that making condoms available to learners in schools promotes positive sexual behavioural outcomes, and Andrzejewski et al. (2019) and Brakman et al. (2017), who report that on-site provision of condoms in schools could decrease risky sexual behaviour. The findings of this study indicate that visual demonstration of sexuality education concepts could enhance learner understanding. Using visual resources to explain different phenomena to young people is a powerful strategy that can enhance their spatial thinking skills (Bobek & Tversky, 2016). Since young people prefer varied learning strategies, Bonjour and Van der Vlugt (2018) call on educators to incorporate visual resources like video and art in their lessons instead of relying only on textbooks. With limited studies available on parental and community acceptance of using video to teach sexuality education concepts in the classrooms, more studies are required to explore potential resistance of the stakeholders, educators’ competencies, learners’ comfort, and other ethical issues regarding video pedagogy in school-based sexuality education programme.

Participants also mentioned on-site school family planning services as a learner-targeted intervention that could improve the effectiveness of sexuality education. While the legal and policy environment in South Africa supports providing learners with school-based SRH services, logistical and resource constraints constitute barriers to its implementation (Khoza et al., 2019). The lack of accessible, youth-friendly, and non-judgemental SRH services in the community reported by the participants make this proposed intervention desirable. There is evidence that on-site school SRH services can successfully provide young people with age-appropriate, convenient and confidential SRH support (Acosta Price, 2016; Keeton et al., 2012; Schelar et al., 2016). School-based SRH services are one of the most effective strategies for providing adolescents in low-resource settings with SRH services (Mason-Jones et al., 2012).

The study revealed that some educators provide sexuality information without demonstration. Such an approach does not equip learners with the critical skills needed to initiate and carry out the desired SRH behavioural changes. Formal demonstration, along with other diverse pedagogical techniques, should be integrated into the sexuality education process (McLain, 2018). Participants advocated that sexuality education lessons should focus on practical application of the acquired knowledge, where concepts taught are demonstrated by educators and subsequently simulated by learners. Hidayati and Pardjono (2018) identify role play as an effective strategy for formal demonstration, and Erturk (2015) highlights that role play is learner-centred and can be used to demonstrate real-life scenarios relevant to the concepts being discussed.

Participants believed that having resident counsellors in their schools would make sexuality education more effective. This proposal is consistent with previous studies which show that school counsellors create a supportive environment that could enhance the SRH skills of learners while promoting positive sexuality education outcomes (Millner & Upton, 2016; Omeje et al., 2012).

Sport, recreational activities, and education are largely interconnected (De Coning, 2018). This opinion correlates with the participants’ suggestion that providing sport and recreational facilities could improve the impact of sexuality education on learners’ SRH. In addition, various studies in diverse settings established that sport and recreational activities enhance young people’s self-efficacy and SRH (Hershow et al., 2015; Barkley et al., 2016; De Coning, 2018).

Social media provides adolescents with attractive platforms for accessing SRH information (UNICEF, 2019) and can be harnessed to enhance the effectiveness of sexuality education. Previous studies have shown that using social media to deliver SRH information to adolescents is effective (Gabarron & Wynn, 2016; Ragsdale et al., 2015). While social media can reach large numbers of young people, Wadham et al. (2019) caution that its information and messages should be high-quality, scientifically accurate, engaging, and evidence-based. Social media is a potential platform from which to access information about SRH care and support services nearby and for collaboration between stakeholders such as parents, schools, and community members.

Setting up help clubs, which are voluntary, community-based non-formal organisations, can complement knowledge acquired at school. Bonjour and Van der Vlugt (2018) and Chirwa-Kambole et al. (2020) agree that help clubs could enhance the effectiveness of sexuality education, leading to positive SRH outcomes among adolescents.

The need for multi-stakeholder collaboration was suggested by the participants, who believed it could address issues such as parental objections, misconceptions, contradictory messages, and the culture of silence about sexuality education. Multi-stakeholder engagement could also promote the acceptance of school-based sexuality education by reluctant community actors. This recommended intervention is supported by a large body of knowledge which indicates that continuous multi-stakeholder collaboration is essential for school-based sexuality education to achieve its health and educational aims among young people (Zulu et al., 2019; Chandra-Mouli et al, 2015; Gudyanga et al., 2019).

Additionally, providing training for parents and community leaders in sexuality education will foster a supportive environment for young people to practise what they learn in the classroom. The findings showed that using opinion leaders and social influencers to endorse and reinforce key SRH messages of school-based sexuality education to relevant stakeholders could promote its acceptance and enhance its effectiveness. Such interventions could be gender-specific, where experienced men will address young boys and experienced women will speak to adolescent girls. This aligns with the report of Pound et al. (2017), which mentions that same-sex group discussions could enhance the impact of sexuality education on adolescents’ SRH.

### Limitations of the Study

The findings of this study were based on the experiences and perspectives of the school-going adolescents; therefore, parental and teachers’ voices were missing from this study. Some focus group interviews took place during the COVID-19 pandemic; therefore, the prevailing anxiety might have influenced how participants spoke about their perspectives and experiences. As a result, interpretations of this study’s findings should take these limitations into consideration.

### Policy Implications

Policy efforts should be focused on addressing the identified learner-centred barriers to effective school-based sexuality education. The provincial DBE should collaborate with the national Department of Health and other stakeholders to devise initiatives that will strengthen the impact of sexuality education on adolescents’ SRH. These initiatives should take into account the identified barriers and the proposed learner-targeted interventions. Besides policy efforts, the DBE should mobilise resources and goodwill of relevant stakeholders develop and implement intervention programmes that will promote access to non-judgemental and youth friendly SRH services by young people in the research setting. Furthermore, sexuality educators’ training should be prioritised to enhance their pedagogical efficiency. The study findings could guide policymakers and relevant stakeholders in programming, implementing and delivering effective school-based sexuality education, leading to improved SRH outcomes for adolescents.

## Conclusions

This study revealed that age disparity, peer pressure, psychological status, exposure to sexualised media content, learners’ attitudes, peer pressure, socio-economic status, lack of recreational facilities, and substance abuse are learner-centred barriers that mitigate the effectiveness of school-based sexuality education in rural areas of King Cethswayo district, South Africa. Based on the shared experiences and views of the participants, we proposed learner-targeted interventions that could overcome these barriers resulting in effective school-based sexuality education and enhanced SRH outcomes among young people. The suggested interventions are multi-stakeholders’ collaboration, advocacy, school condom distribution, awareness campaigns, visual presentations, using guest health-care professionals, on-site family planning services, formal demonstrations, school counselling services, sport activities, using social media platforms, and forming help clubs. In addition, the intervention should incorporate pre-service, continuous in-service training and support for the educators. We argue that reviewing and revising existing policies on sexuality education in the light of the study findings could enhance the programming, implementation and delivery of school-based sexuality education, leading to positive SRH outcomes among adolescents.
